# Porous SnO_2_/C Nanofiber Anodes and LiFePO_4_/C Nanofiber Cathodes with a Wrinkle Structure for Stretchable Lithium Polymer Batteries with High Electrochemical Performance

**DOI:** 10.1002/advs.202001358

**Published:** 2020-07-19

**Authors:** O. Hyeon Kwon, Jang Hyeok Oh, Bobae Gu, Min Su Jo, Se Hwan Oh, Yun Chan Kang, Jae‐Kwang Kim, Sang Mun Jeong, Jung Sang Cho

**Affiliations:** ^1^ Department of Energy Convergence Engineering Cheongju University Cheongju Chungbuk 28503 Republic of Korea; ^2^ Department of Engineering Chemistry Chungbuk National University Cheongju Chungbuk 361‐763 Republic of Korea; ^3^ Department of Materials Science and Engineering Korea University Anam‐Dong, Seongbuk‐Gu Seoul 136‐713 Republic of Korea; ^4^ Department of Chemical Engineering Chungbuk National University Cheongju Chungbuk 361‐763 Republic of Korea

**Keywords:** lithium‐ion batteries, nanofibers, stretchable batteries, stretchable gel polymer electrolytes, wrinkle structure

## Abstract

Stretchable lithium batteries have attracted considerable attention as components in future electronic devices, such as wearable devices, sensors, and body‐attachment healthcare devices. However, several challenges still exist in the bid to obtain excellent electrochemical properties for stretchable batteries. Here, a unique stretchable lithium full‐cell battery is designed using 1D nanofiber active materials, stretchable gel polymer electrolyte, and wrinkle structure electrodes. A SnO_2_/C nanofiber anode and a LiFePO_4_/C nanofiber cathode introduce meso‐ and micropores for lithium‐ion diffusion and electrolyte penetration. The stretchable full‐cell consists of an elastic poly(dimethylsiloxane) (PDMS) wrapping film, SnO_2_/C and LiFePO_4_/C nanofiber electrodes with a wrinkle structure fixed on the PDMS wrapping film by an adhesive polymer, and a gel polymer electrolyte. The specific capacity of the stretchable full‐battery is maintained at 128.3 mAh g^−1^ (capacity retention of 92%) even after a 30% strain, as compared with 136.8 mAh g^−1^ before strain. The energy densities are 458.8 Wh kg^−1^ in the released state and 423.4 Wh kg^−1^ in the stretched state (based on the electrode), respectively. The high capacity and stability in the stretched state demonstrate the potential of the stretchable battery to overcome its limitations.

## Graphical abstract

A novel approach toward high‐performance stretchable batteries that applies 1D nanofiber active materials, stretchable gel polymer electrolyte, and an electrode with wrinkled structure is successfully demonstrated. The stretchable full‐battery exhibits high capacity, high energy density, and notable stability even in the stretched states.

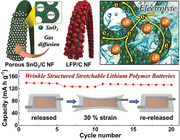

## 1 Introduction

In today's world of swift technological expansion, there is an inordinate obligation to achieve advancement in the field of stretchable energy storage system.^[^ ^1^, ^2^, ^3^, ^4^ ^]^ These stretchable devices can provide combined advantages in terms of superior electrochemical features as well as mechanical sturdiness. As a result, they can be used in various fields such as biomedical grafting, smart textiles, and stretchable electronics.^[^ ^1^, ^2^, ^3^, ^4^ ^]^ Greater Coulombic efficiency and enhanced energy density of the lithium‐ion battery (LIB) makes it the most attractive candidate among them.^[^ ^5^, ^6^, ^7^, ^8^ ^]^ Technology has become an integral part of our life due to which the importance of stiff and hefty batteries is declining, and the focus is gradually shifting to stretchable batteries. Moreover, LIBs consisting of a combustible organic liquid electrolyte, which may be hazardous due to leakages and thermal runaway.^[^ ^9^, ^10^, ^11^, ^12^ ^]^ Due to these limitations, there is a great demand for developing components that can provide maximum stretchability to the LIBs while maintaining their safety.^[^ ^13^, ^14^, ^15^, ^16^ ^]^

To enable fabrication of stretchable batteries, either the components must be replaced with stretchable materials, or the existing materials must be designed with stretchable structures and stretchable electronics that can accommodate mechanical stress while maintaining percolation thresholds. Recent development of the battery systems for realizing stretchability includes wrinkled design, buckled and elastic scaffold, and wavy structure using elastomeric materials, so as to reduce the loss of conductivity during deformation.^[^ ^17^, ^18^, ^19^, ^20^ ^]^ In addition, there is a configuration of a spiral coil spring that is used to prevent the penetration of air and moisture.^[^ ^21^, ^22^, ^23^ ^]^ However, this type of stretchable LIBs exhibit a degree of stretchability that limits their stability when strained. Because the internal structure is easily changed by stretching, 1D nanofiber‐shaped electrodes may help overcome this limited stability.

In this study, nanofiber‐shaped unique metal compound electrodes reinforced with C were prepared by electrospinning for stretchable LIBs. For the anode, porous SnO_2_/C nanofibers with uniformly defined mesopores were prepared. To obtain these SnO_2_/C nanofibers, PS nanobeads were used as a pore‐forming agent in the PVA/SnCl_4_ solution, followed by electrospinning and heat treatment. By adding the size‐controlled polystyrene (PS) nanobeads in a spinning solution, it was possible to obtain nanofibers with a uniform pore size and distribution in the nanostructure after decomposition. The porosity of these electrodes leads to better electrolyte penetration and Li^+^ ion diffusion during cycling. Additionally, the numerous mesopores in the structure and carbon matrix facilitate the accommodation of the huge volume expansion of the SnO_2_ active materials during repeated cycling. Further, electrospinning of LFP/PAN(polyacrylonitrile) solution was carried out, followed by thermal treatment, to obtain LFP/C nanofibers. The electrospun LFP forms an effective conductive C network with a uniform distribution of the C layers, and shortens the diffusion path for excellent cycling performance. The excellent properties of SnO_2_/C nanofiber and LFP/C nanofiber electrodes with a high operation voltage (3.3 V) are beneficial in achieving high energy density without the use of an aqueous electrolyte. The high operation voltage of the stretchable battery increases energy density because that is the requirement of voltage and capacity of batteries, whereas the stretchable batteries that use an aqueous electrolyte are limited to an operation voltage of less than 2.5 V.^[^ ^24^, ^25^, ^26^, ^27^, ^28^ ^]^ We demonstrated higher stability and conductivity of the electrodes in the strained wrinkle structure using nanofiber active materials. The formation and mechanism of such unique hybrid nanofibers, and their electrochemical properties for stretchable lithium polymer batteries were investigated in detail.

## 2 Results and Discussion

**Figure 1:**
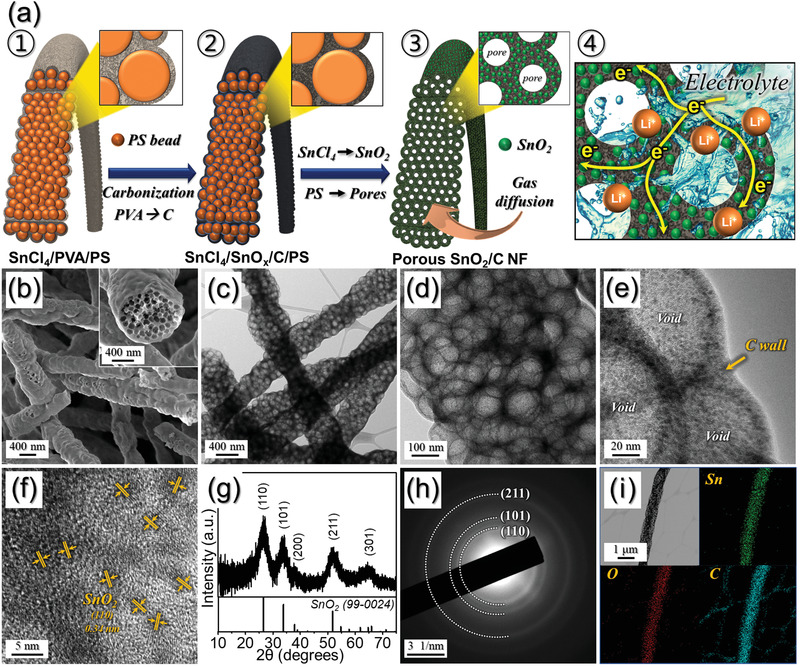
a) Formation scheme of the porous SnO_2_/C NF as anodes, b) FE‐SEM, c–f) TEM images, g) XRD pattern, h) SAED pattern, and i) elemental mapping images of the porous SnO_2_/C NF.

**Figure 2:**
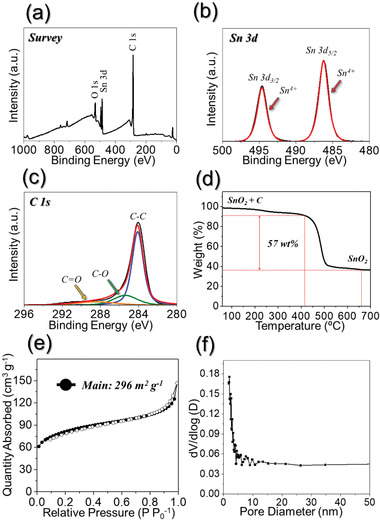
Characteristics of the porous SnO_2_/C NF. a) Survey XPS spectrum, b) Sn 3d XPS spectrum, c) C 1s XPS spectrum, d) TGA curve, e) N_2_ adsorption and desorption isotherms, and f) pore size distribution.

**Figure 3:**
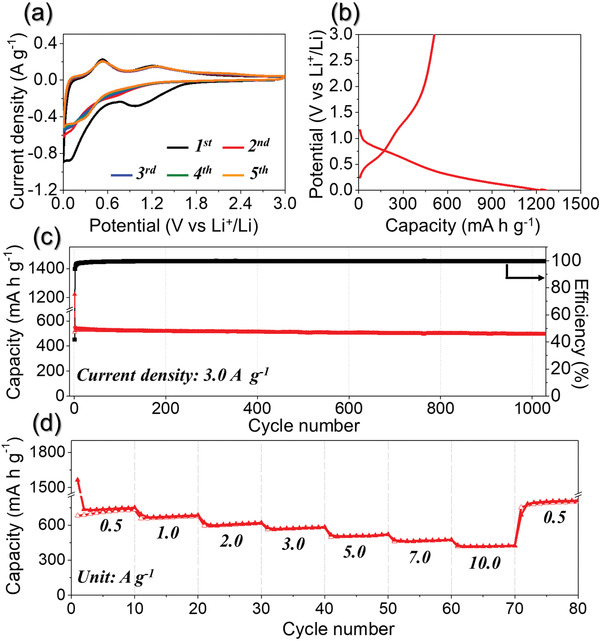
Electrochemical properties of the porous SnO_2_/C NF. a) CV curves, b) initial discharge–charge profile, c) cycling performance at a current density of 3.0 A g^−1^, and d) rate performance.

**Figure 4:**
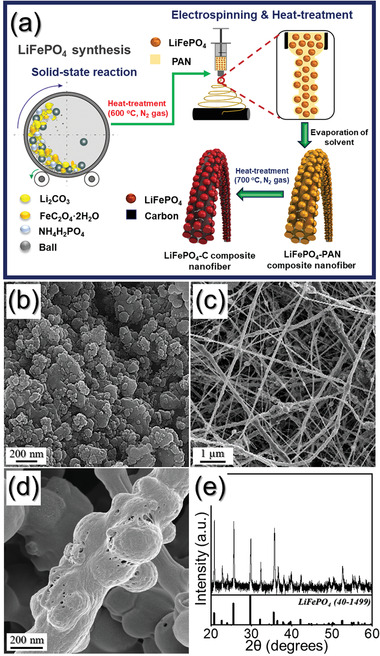
a) Scheme of the formation strategy for the LFP/C NF as cathodes, b) FE‐SEM image of the LFP powders after solid‐state reaction, c) LFP/PAN composited nanofibers obtained after electrospinning, d) LFP/C NF obtained after heat‐treatment, and its e) XRD pattern.

One of the key challenges for stretchable energy storages is the improvement of their energy density, which is strongly related to the electrode materials. Until now, the active materials for electrodes focused on conventional materials such as LiCoO_2_ (LCO), LiFePO_4_ (LFP), and LiMn_2_O_4_ (LMO) cathodes assembled with Li_4_Ti_5_O_12_ (LTO) and graphite anodes. In this study, the high‐energy stretchable polymer LIBs were prepared by assembling porous SnO_2_/C nanofibers (porous SnO_2_/C NF) anodes and LFP/C NF cathodes. For this, unique structured metal compound electrodes reinforced with C were prepared by electrospinning and/or solid‐state reaction processes. For the anode, porous SnO_2_/C NF was prepared by electrospinning and subsequent heat‐treatment. The details of formation mechanism of the porous SnO_2_/C NF with uniformly distributed mesopores as anode materials are described in **Figure** **1**a. For this, the precursor nanofibers composed of Sn salt, PVA, and uniformly distributed PS nanobeads (100 nm) were obtained by electrospinning process (Figure 1a‐①). The as‐spun fibers were shown in Figure S1, Supporting Information. The size‐controlled PS nanobeads of 100 nm as pore generator were prepared by emulsifier‐free emulsion polymerization method (Figure S2, Supporting Information). During the heat‐treatment of as‐spun nanofibers at 350 °C, PVA was carbonized and it formed a nanofiber matrix reinforced with C as shown in Figure 1a‐②. PS nanobeads in the composite were selectively decomposed into gaseous products, thus creating uniform well‐defined mesopores in the structure. Additionally, Sn salt in the as‐spun fibers was converted into SnO_2_ (Figure 1a‐③).^[^ ^29^, ^30^ ^]^ During the heat‐treatment process, the C surrounding Sn salt inhibits the growth of SnO_2_ crystals and the pores enable the diffusion of gas into the structure for the homogeneous oxidation of Sn salt. Therefore, the porous SnO_2_/C NF containing numerous mesopores was obtained, as shown in Figure 1b. Although, SnO_2_ has high theoretical capacity of 782 mAh g^−1^, the large volume variation during repeated cycles induced capacity fading.^[^ ^31^, ^32^, ^33^ ^]^ However, the mesopores in such structures and the C surrounding SnO_2_ could accommodate the stress induced by volume variation. Additionally, the C present in the structure contributes to the high electrical conductivity, which provides conductive channels and multiple pathways for the electron (Figure 1a‐④). Moreover, the porous structure facilitates efficient infiltration of the electrolyte into the structure, which promotes the electron transfer kinetics; this consequently leads to an improved Li^+^ ion diffusion rate. The porous SnO_2_/C NF has a uniform thickness with a mean diameter of 800 nm. In particular, it contains numerous well defined mesopores (diameter = 80 nm) uniformly distributed in the structure as shown in the cross sectioned image (inset of Figure 1b) and TEM images (Figure 1c–e). In Figure 1e, the thickness of the carbon shell of pores was measured as 9 nm, in which SnO_2_ nanocrystals with a size less than 3 nm were homogeneously surrounded by the C matrix. The complete conversion of Sn salt into SnO_2_ nanoparticles occurred, due to which the lattice fringes separated by 0.34 nm corresponding to the (110) crystal plane of SnO_2_ are observed in Figure 1f.^[^ ^34^ ^]^ The XRD and SAED patterns in Figure 1g,h also show the (110), (101), and (211) crystalline planes of SnO_2_, further proving the successful formation of SnO_2_.^[^ ^35^, ^36^ ^]^ The crystallite size of this SnO_2_ phase was calculated to be 2.5 nm using Scherrer's equation. The elemental mapping images for the Sn, O, and C elements in the porous SnO_2_/C NF in Figure 1i, indicate the uniform distribution of SnO_2_ nanocrystals in C matrix.

The chemical state and molecular environment of the porous SnO_2_/C NF were characterized by X‐ray photoelectron spectroscopy (XPS), shown in **Figure** **2**a–c. In Figure 2a, the XPS spectrum of the porous SnO_2_/C NF shows peaks corresponding to Sn, O, and C signals. In the Sn 3d XPS spectrum in Figure 2b, the peaks of Sn 3d_5/2_ at 486.3 eV and Sn 3d_3/2_ at 494.6 eV corresponding to Sn^4+^ were observed, which are attributed to the formation of SnO_2_.^[^ ^37^, ^38^, ^39^, ^40^ ^]^ The C 1s XPS spectrum in Figure 2c includes peaks corresponding to C—C, C—O, and C=O bonds at 284.1, 285.4, and 288.4 eV, respectively.^[^ ^41^, ^42^, ^43^, ^44^ ^]^ The highest intensity of the C—C peak among the three peaks confirms the carbonization of PVA during heat‐treatment. Thermogravimetric analysis (TGA) was carried out in order to confirm the amount of C decomposed by PVA in the structure, as shown in Figure 2d. Evaporation of adsorbed water molecules was responsible for the weight loss at around 250 °C. The following steep weight loss between 420 and 500 °C was associated with the combustion of C, and amounted for approximately 57 wt%. The Brunauer–Emmett–Teller (BET) surface area of the porous SnO_2_/C NF was 296 m^2^ g^−1^ (Figure 2e). The isotherms indicated the presence of micropores (diameter < 2 nm) originating due to the carbonization of PVA and mesopores below 50 nm diameter attributed to the space formed due to the decomposition of PS nanobeads during heat‐treatment. This high BET surface area is attributed to the unique nanostructure of the porous SnO_2_/C NF, micropores, mesopores, and the carbon content of the structure. The Barrett–Joyner–Halenda pore size distribution of the porous SnO_2_/C NF shown in Figure 2f suggests that the majority of the pores have sizes below 5 nm.

The electrochemical properties of the porous SnO_2_/C NF used as anodes are shown in **Figure** **3**. The CVs of the porous SnO_2_/C NF for the first five cycles at a scan rate of 0.1 mV s^−1^ in the potential range of 0.001–3.0 V are shown in Figure 3a. During the first cathodic step, two reduction peaks were observed at approximately 0.8 and 0.2 V, probably due to the conversion of SnO_2_ to metallic Sn and subsequent alloying reaction of metallic Sn with Li^+^ ion.^[^ ^45^, ^46^, ^47^, ^48^, ^49^ ^]^ The first peak at 0.8 V was mainly associated with the formation of both metallic Sn nanograins and amorphous Li_2_O through the reduction of SnO_2_.^[^ ^45^, ^46^, ^47^, ^48^, ^49^ ^]^ The second peak at 0.2 V was attributed to the alloying reaction between Li and Sn, which formed Li*_x_*Sn alloys.^[^ ^45^, ^46^, ^47^, ^48^, ^49^ ^]^ In the anodic scan, two peaks located at 0.5 and 1.2 V were observed, attributed to the de‐alloying reaction of Li_4.4_Sn and oxidation of Sn into SnO_2_.^[^ ^45^, ^46^, ^47^, ^48^, ^49^ ^]^ In the subsequent cycles, two redox peak pairs appeared at 0.36 V/0.5 V and 0.5 V/1.2 V, which corresponded to the redox reaction of SnO_2_.^[^ ^45^, ^46^, ^47^, ^48^, ^49^ ^]^ The good overlapping of the CV curves from the third cycle onward revealed good reversibility of the electrochemical reactions during cycles. The discharge–charge profile of the porous SnO_2_/C NF at a high current density of 3.0 A g^−1^ was shown in Figure 3c. The initial discharge and charge capacities of the porous SnO_2_/C NF were 1223 and 510 mAh g^−1^, respectively, and the calculated initial columbic 41%. The discharge capacities of the 2nd and 1000th cycles were 556 and 495 mAh g^−1^, respectively, and the capacity retention measured from the second cycle was 89%. The porous SnO_2_/C NF anodes could accommodate the large volume variation during repeated lithium alloying de‐alloying and decrease Li^+^ diffusion length, thus leading to the superior cycling stability even at the high current density of 3.0 A g^−1^.

The rate performance of the porous SnO_2_/C NF is shown in Figure 3d, in which the current density increases in a step‐wise manner from 0.5 to 10 A g^−1^, with 10 cycles being performed at each step. The nanofibers exhibited good rate performance. The final discharge capacities of the porous SnO_2_/C NF were 750, 685, 620, 584, 521, 475, and 425 mAh g^−1^ at respective current densities of 0.5, 1.0, 2.0, 3.0, 5.0, 7.0, and 10.0 A g^−1^. Subsequently, the capacity recovered to 798 mAh g^−1^ when the current density was returned to 0.5 A g^−1^. The excellent rate property of the porous SnO_2_/C NF is attributed to its unique nanostructure. The efficient penetration of the liquid electrolyte into the electrode during cycling was possible due to its porous structure, which promoted the electrochemical reaction. Additionally, the C matrix surrounding SnO_2_ contributed to the high electrical conductivity of the electrode, which facilitated fast electron transfer by improving the electrical contact between the active sites of the SnO_2_ nanoparticles and the electrode.

1D structured cathode materials were also prepared by continuous solid‐state reaction and electrospinning processes and subsequent heat treatment (**Figure** **4**a). For this, LFP powders with a mean size of 150 nm were first obtained by solid‐state reaction, carrying by N_2_ gas at 600 °C for 10 h during heating as shown in Figure 4b. Subsequently, the obtained LFP powders were added into the DMF solution with PAN in order to perform electrospinning, which resulted in LFP/PAN composited nanofibers shown in Figure 4c. After the heat treatment, PAN was carbonized, resulting in nanofibers composed of carbon matrix embedded with LFP particles. The LFP/C nanofibers obtained after heat‐treatment process are shown in Figure 4d. The 1D nanofiber network of LFP/C electrode could enhance the stretchability and electrochemical properties, resulting in a short diffusion distance of Li^+^ ions. Figure 4e shows the result of fitting the XRD pattern for LFP/C NF after heat treatment. All diffraction peaks are indexed according to JCPDS file no. 40–1499 of olivine crystal structure.^[^ ^50^, ^51^ ^]^ No impurity phases were present in the peak patterns of the materials in patterns of the LFP, and the peak profiles are narrow indicating a well‐crystallized phase. Figure S3, Supporting Information, shows the electrochemical properties of LFP/C NF as cathode materials. The CV curves of 1, 2, and 5 cycles are shown in Figure S3a, Supporting Information, at a scan rate of 0.1 mV s^−1^ within a potential window of 2.0–4.5 V (vs Li/Li^+^). The three cycles are almost superimposed, indicating good electrochemical reversibility, and high oxidation. Reduction peaks imply good electronic conductivity. In addition, redox peak pairs shown in the curves are observed between 3.29 and 3.56 V, which is due to the redox reaction of Fe^2+^/Fe^3+^.^[^ ^52^, ^53^, ^54^, ^55^ ^]^ The small voltage difference (∆*V*) between reduction voltage and oxidation voltage means a low cell resistance^[^ ^56^, ^57^ ^]^, which is beneficial for a high electrochemical performance. Figure S3b, Supporting Information, shows the initial charge and discharge curves of LFP/C NF cell at 0.5 C. The specific discharge capacity is 155 mAh g^−1^ at 0.5 C. Moreover, the curves show high electrochemical reversibility with little loss of capacity in the charge and discharge curves. The LFP/C NF cell remains stable at 136.7 mAh g^−1^ for over 200 cycles. With an initial capacity of 155 mAh g^−1^, in all cycles, the Coulombic efficiency was more than 98%, and the discharge capacity retention rate was about 88.2%.

**Figure 5:**
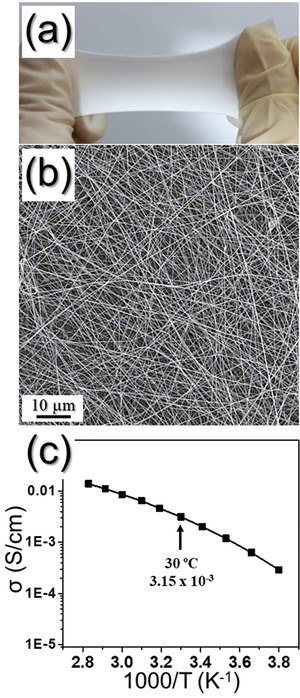
a) Photograph of the stretched PVdF‐HFP sheet, b) FE‐SEM image, and c) ionic conductivity as function of temperature of the PVdF‐HFP gel polymer electrolyte.

The PVdF‐HFP matrix used as the gel polymer electrolyte was prepared by electrospinning process as shown in **Figure** **5**. Figure 5a shows the high stretchability and the mechanical strength of the PVdF‐HFP matrix at 30% stretching. In terms of tensile strength, the average stress of the electrospun PVdF‐HFP matrix was 28.2 MPa (0.8 kgf) (Figure S4, Supporting Information). These properties of PVdF‐HFP matrix make it suitable for use as gel polymer electrolytes to fabricate a stretchable battery. The morphology of the electrospun PVdF‐HFP membrane is shown in the FE‐SEM image in Figure 5b. The fibers were homogeneously interconnected with an average diameter of 1 µm, resulting in a 500 µm thick sheet and a relatively smooth and straight surface. Such interconnection of the fibers imparts sufficient mechanical strength to the membrane for stretching. The presence of the fully interconnected micropores in the matrix makes it ideal for application as a host membrane for the preparation of gel polymer electrolytes. In Figure 5c, the ionic conductivity of the gel polymer electrolyte is shown as a function of temperature. The ionic conductivity gradually increased with increasing temperature in the measured temperature range, and achieved a value of 3.15 × 10^−3^ S cm^−1^ at 30 °C. The high porosity of the electrospinning matrix and, more importantly, the interconnectivity of micron‐sized pores assist the easy migration of lithium ions, resulting in a high ionic conductivity even at lower temperatures, for example, 2.89 × 10^−4^ S cm^−1^ at −10 °C.

**Figure 6:**
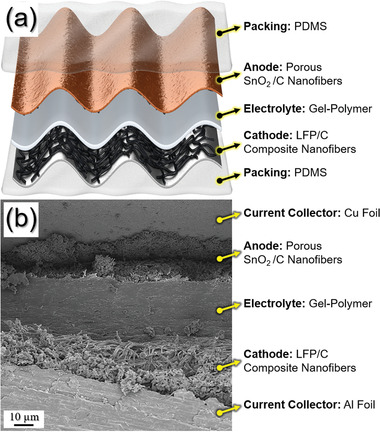
a) Scheme illustration of the device for stretchable full‐cell batteries assembled with integrating PDMS@LFP/C NF@GPE@SnO_2_/C NF@PDMS based on wrinkle structure and b) FE‐SEM image of the cross‐sectioned stretchable full‐cell batteries.

The stretchable full‐cell battery is assembled by integrating PDMS@LFP/C NF@GPE@SnO_2_/C NF@PDMS based on wrinkle structure (**Figure** **6**a). Currently, stretchable battery systems have bulk electric scaffold and wavy structure.^[^ ^58^ ^]^ However, these types of stretchable batteries have limited stability during stretching, which points to a disadvantage of stretchability. To overcome this, nanofiber‐shaped electrodes with a wrinkle structure are a solution to the internal structure that is easily changed due to stretching. In this system, each arched anode, arched gel polymer electrolyte (GPE), and arched cathode is packaged by an elastic PDMS film, which constitute the battery in Figure 6a. The extension of the waves of the anode, GPE, and cathode crucially leads to the stretchability of the cell. During the repeated release and stretched states, the valleys of the wavy electrodes were fixed on the PDMS wrapping film by using adhesive polymer to prevent movement of the electrodes position. In the both electrodes, the carbon layer of the electrospun LFP forms an effective conductive carbon network with uniform distribution. In the anode, the porosity promotes electrolyte penetration and Li^+^ ion diffusion. PDMS filled in curved wave structures can be reversibly tensioned and deformed. In addition, electrospun PVdF‐HFP exhibits high mechanical strength and electrode stability, and is used as a GPE and a solid electrolyte in LIBs. It also has high porosity, elasticity, and ionic conductivity. In order to verify a good interface between the electrodes and electrolyte, which is crucial for the electrochemical performance, cross‐sectioned image of the stretchable full‐cell batteries was examined by FE‐SEM in Figure 6b. The stretchable PDMS@LFP/C NF@GPE@SnO_2_/C NF@PDMS full‐cell batteries show good interfacial contact between the electrodes, electrolyte, and current correctors. No apparent delamination between the nanofiber electrodes and GPE has been observed, indicating good interfacial bonding during the 30% strain.

**Figure 7:**
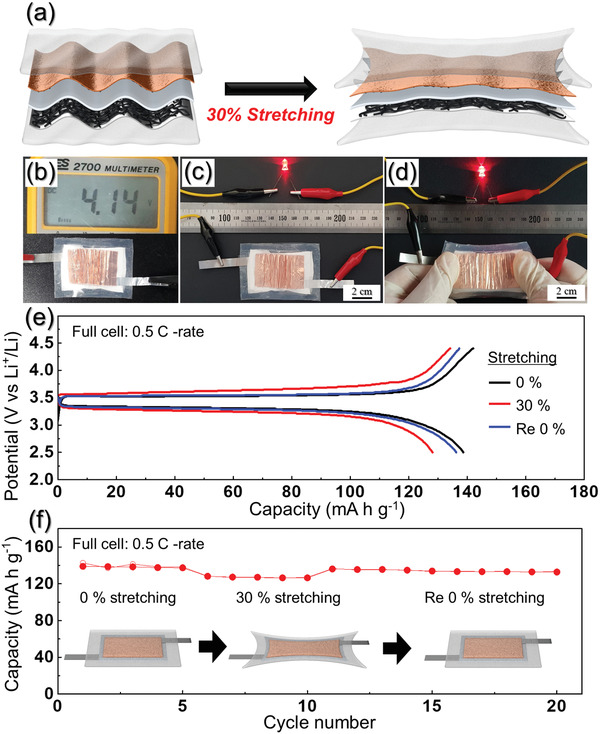
a) Schematic of the stretchable full‐cell battery in released and stretched (30% strain) states, b) Digital photographs of the full‐cell battery and its voltage output, and c,d) an LED lit by the stretchable full cell in its released (c) and stretched (d) states, e) charge–discharge profile, and f) cycling performance of the full‐cell battery in released and stretched states (0–30%) at 0.5 C.

**Figure** **7**a shows schematics of stretchable full‐cell battery in released and stretched states (0–30%). In the conventional electrode using flat‐type metal current collector, the active electrode layer can crack due to lack of elastic deformation.^[^ ^59^ ^]^ However, the designed wavy electrode on PDMS wrapping film can be stretched without cracks due to its wrinkle structure and the elasticity of PDMS. The voltage charging profile of the stretchable full‐cell is displayed in Figure 7b. After charging to 4.5 V, the voltage rapidly dropped to 4.14 V and slowly reached a flat voltage (3.55 V) in the charge curve (Figure 7e). The notable aspect of the voltage profile is that the stretchable cell is capable of high voltage charging to achieve high energy density without using aqueous electrolyte. The battery connected to a red light‐emitting diode (LED) is shown in Figure 7c,d at released and stretched status, respectively. The stretchable battery continuously powered the LED even at the stretched state of 30%. Figure 7e,f show the electrochemical performances for the stretchable battery at 0.5 C using GPE, porous SnO_2_/C NF, and LFP/C NF electrodes. Although the voltage gap between charge curve and discharge curve slightly increased because of the strain on the electrode, the charge–discharge curves (Figure 7e) of the stretchable battery at released and stretched states reached discharge capacities of 138.6 and 128.3 mAh g^−1^, respectively, indicating nearly constant capacity. The energy densities are 458.8 Wh kg^−1^ in released state and 423.4 Wh kg^−1^ in stretched state (based on the electrode), respectively. The stretchable full‐cell battery introduced in this study showed the highest energy densities as compared to the other stretchable batteries reported previously (Table S1, Supporting Information). In addition, in the re‐released states, the discharge capacity reached 136.4 mAh g^−1^. This enhanced performance for the stretchable battery under both the released and stretched status is due to the good contact between gel electrolyte and nanofiber electrodes, and the robust adhesion by adhesive polymer between the wrinkled current collector and the elastic PDMS wrapping film.

Figure 7f shows the cycle properties of the stretchable PDMS@LFP/C NF@GPE@SnO_2_/C NF@PDMS full‐cell in released and stretched states (0–30%) at 0.5 C. The first charge–discharge cycle for the stretchable full‐cell was performed at the released state. Then, the cell was run alternatively at 30% strain and re‐released states for 20 cycles. The specific capacity was maintained at 138.3 mAh g^−1^ during the re‐released state and at 127.0 mAh g^−1^ during the 30% strain, after 5 cycles, exhibiting an excellent capacity retention of 99.6% and 98.9% in released and stretched states, respectively. In addition, it almost overcame the initial capacity of the first released state in the re‐released states, which indicates the excellent stability of the stretchable full‐cell. These results demonstrate that stretchable full cell based nanofiber electrodes and gel polymer electrolyte are an appropriate power source for various stretchable electronic devices.

## 3 Conclusion

We have successfully developed a stretchable LIB based on a 1D nanofiber electrode design and a wrinkle structure electrode. The electrospun nanofibers were prepared to ensure the stability of stretchable batteries. Both electrodes formed a conductive carbon network with uniformly distributed carbon layers. In addition, the porous SnO_2_/C NF, used as anodes, promoted electrolyte penetration and Li^+^ ion diffusion through micro‐ and mesopores. These nanofibers exhibited high stability and conductivity in wavy structure electrodes for stretchable batteries, and had a reduced capacity loss during stretching. A stretchable PVdF–HFP gel polymer electrolyte was first used as electrolyte in stretchable batteries, which could be stuck to nanofiber electrodes to assure an enhanced ion contact, especially during dynamic motions. High electrochemical performance and good stability for the stretchable full‐cell at released and stretched states were demonstrated. High discharge capacities of 138.6 mAh g^−1^ in released state and 128.3 mAh g^−1^ in stretched state could be achieved for the stretchable battery with high energy density (458.8 Wh kg^−1^). The strategy introduced in this work can be applied for the fabrication of highly integrated and interconnected SnO_2_/C and LFP/C composite nanofiber electrodes for a variety of applications, including stretchable batteries. Moreover, the stretchable battery concept of wrinkle structure nanofiber electrodes, stretchable gel electrolyte, elasticity of PDMS wrapping film, and fixation of wrinkled electrode by adhesive polymer can be extended to new electronic devices, thus paving the way for a promising future.

## 4 Experimental Section

##### Synthesis of SnO_2_/C Composite Nanofibers with Uniform Mesopores as Anode Materials

Porous SnO_2_/C composite nanofibers with uniformly distributed mesopores were prepared by electrospinning and subsequent heat treatment. First, precursor fibers were obtained by electrospinning process. The spinning solution was prepared by dissolving 2.0 g of Tin(IV) chloride pentahydrate (SnCl_4_·5H_2_O, Kanto, 98.0%) and 1.5 g of polyvinyl alcohol (PVA 2000, Kanto) in 15.0 mL ethyl alcohol (EtOH, Duksan, 99.9%). Then, 15.0 mL of an aqueous suspension containing PS nanobeads (1.5 g) as a template was added to the above solution, and was stirred vigorously overnight. The PS nanobeads with a diameter of 100 nm were prepared by emulsion polymerization method, which is described in the authors’ previous work.^[^ ^60^, ^61^, ^62^, ^63^ ^]^ The prepared spinning solution was loaded into a plastic syringe equipped with a 23‐gauge stainless‐steel nozzle, ejected at a flow rate of 1.0 mL h^−1^, and electrospun onto a drum collector. During electrospinning, the distance between the tip and the collector was maintained at 12 cm, while the rotation speed of the drum was maintained at 150 rpm. A voltage of 20 kV was applied between the collector and the syringe tip. The resulting as‐spun fibers composed of Sn salt, PVA, and PS nanobeads, were stabilized at 100 °C under air for 24 h. Subsequently, the composite fibers were thermally treated at 350 °C at a heating rate of 1 °C min^−1^ under N_2_ atmosphere for 1 min. This continuous process resulted in SnO_2_/C composite nanofibers with uniformly distributed mesopores; these nanofibers were denoted as “porous SnO_2_/C NF.”

##### Synthesis of LiFePO_4_/C Composite Nanofibers as Cathode Materials

LFP powders were synthesized by a solid‐state reaction from the precursors (Li_2_CO_3_, FeC_2_O_4_·2H_2_O, and NH_4_H_2_PO_4_, 99%, Aldrich) in stoichiometric quantities. First, the precursors were mixed by high‐energy ball milling in a hardened steel vial with zirconia balls at 20 °C for 15 h under argon atmosphere. Then they were subjected to heat treatment at 600 °C at a heating rate of 5 °C min^−1^ for 10 h under nitrogen atmosphere. The resulting spherical LFP powders after solid‐state reaction were reinforced with carbon as a 1D structure with the help of electrospinning. For this, the spinning solution was prepared by adding 5.0 g LFP microspheres obtained by solid‐state reaction and 5.0 g polyacrylonitrile (PAN, Polysciences, *M*w: 150 000) in *N*,*N*‐dimethylformamide (DMF, Aldrich, 99.8%). The prepared spinning solution was loaded into a plastic syringe equipped with a 21‐gauge stainless‐steel nozzle. It was released at a flow rate of 1 mL h^−1^ on the collector drum rotating at 180 rpm. During the electrospinning process, the distance between the tip and the collector was fixed at 15 cm. The applied voltage between the collector and the needle tip was 20 kV. The electrospun fibers were stabilized at 100 °C in an air atmosphere for 6 h. The stabilized fibers were heat‐treated at 700 °C at a heating rate of 5 °C min^−1^ for 3 h in N_2_ gas atmosphere. This process resulted in LFP/C composite nanofibers (LFP/C NF), which were applied as cathode materials.

##### Synthesis of Gel Polymer Electrolyte

Microporous matrix of PVdF‐HFP (Kynar 2801) was prepared by means of electrospinning as per the procedure standardized in the authors’ previous study.^[^ ^64^, ^65^ ^]^ A 16 wt% solution of PVdF‐HFP in a mixed solvent of acetone and *N*,*N*‐dimethylacetamide (7/3, w/w) was electropsun by applying an electric voltage of 18 kV. A thin film of ≈1 µm thickness was collected on an aluminum foil. The electrospun membrane was vacuum‐dried at 60 °C for 12 h before further use. The GPE was prepared by immersing the electrospun membrane in 1 m solution of lithium hexafluorophosphate (LiPF_6_) in ethylene carbonate (EC)/diethyl carbonate (DEC) (1:1, v/v) with 200% uptake. Activation of the membrane to prepare the GPE was carried out in an argon‐filled glove box under a moisture level of less than 10 ppm.

##### Characterization

The morphologies were observed using field‐emission scanning electron microscopy (FE‐SEM, Zeiss, ULTRA Plus) and high‐resolution transmission electron microscopy (HR‐TEM, JEOL, JEM‐2100F, working voltage = 200 kV). The crystal structure was investigated by X‐ray diffraction (XRD, Bruker AXS, D8 Discover with GADDS) using Cu K_*α*_ radiation (*λ* = 1.5418 Å). Structures of carbonaceous materials in the composite were characterized at 20 °C via Raman spectroscopy (Jobin Yvon LabRam, HR800, excitation source = 514 nm He–Ne laser). XPS (Thermo Scientific K‐Alpha) with a focused monochromatic Al K_*α*_ at 12 kV and 20 mA was used to analyze compositions of the nanofibers. Surface area of the sample was measured using the BET method with N_2_ as the adsorbate gas (TriStar 3000 gas adsorption analyzer, Micromeritics, USA). TGA was performed using a Pyris 1 TGA (PerkinElmer, KBSI) within temperature range of 25–700 °C at a heating rate of 10 °C min^−1^ in air.

##### Electrochemical Measurements

For the half‐cell test using 2032‐type coin cells, the anode was prepared by mixing 70 wt% porous SnO_2_/C NF active material, 20 wt% carbon black, and 10 wt% sodium carboxymethyl cellulose. Li metal and GPE were used as the counter electrode and the electrolyte, respectively. The charge/discharge characteristics of the samples were investigated by cycling over a potential range of 0.001–3.0 V at various current densities. Cyclic voltammograms (CVs) measured at a scan rate of 0.01 mV s^−1^ were examined. The size of the negative electrode containing the tin fibers was 0.785 cm^2^ and the mass loading was approximately 0.91 mg cm^−2^. In this study, the capacities of the samples were calculated based on the total mass of the prepared sample. Electrochemical impedance spectra were obtained by performing alternating‐current electrochemical impedance spectroscopy (EIS, ZIVE SP1) over a frequency range of 0.01 Hz to 100 kHz at a potential of 1 mV. The electrochemical performances of the LFP/C NF as the cathode material in LIBs were examined using the 2032‐type coin cells. The prepared LFP/C NF was used as the working electrode and was composed of 80 wt% active materials and 10 wt% carbon black (Super‐P) as the conductive materials, with 10 wt% poly(vinylidene fluoride) (PVdF) as the binder on the aluminum foil. The Li metal and GPE were once again used as the counter electrode and the electrolyte, respectively. The charge/discharge characteristics of the samples were investigated by cycling over a potential range of 2.0–4.5 V. The size of the positive electrode containing the LFP/C NF was 0.785 cm^2^ and the mass loading was 5.3 mg cm^−2^. Before fabrication of the stretchable full‐cell with LFP/C NF cathode, the porous SnO_2_/C NF electrode was pre‐lithiated to decrease the irreversible capacity loss occurring during the cycling of the half‐cell assembly between 0.001 and 3.0 V. For the stretchable full‐cell test, the slurry coated copper and aluminium foils were subjected to a unidirectional (UD) compressive stress using a molding arrangement as shown in Figure S5, Supporting Information. This subsequently resulted in equally spaced wrinkled‐type electrodes, which were stacked over each other to form a full cell. GPE was used as the stretchable electrolyte. The electrochemical properties of the pouch‐type full‐cells consisting SnO_2_/C@GPE@LFP/C were examined at 2.72 mA in the voltage window of 2.0–4.5 V in a dry room with a dew point −60 °C.

## Conflict of Interest

The authors declare no conflict of interest.

## Supporting information

Supporting Information
