# Supplementary material for: Porous SnO2/C Nanofiber Anodes and LiFePO4/C Nanofiber Cathodes with a Wrinkle Structure for Stretchable Lithium Polymer Batteries with High Electrochemical Performance
Source: Adv Sci (Weinh). 2020 Jul 19;7(17):2001358. doi: 10.1002/advs.202001358 (PMC7507473; doi:10.1002/advs.202001358)
Supplement: Supplementary file 1 — Supporting Information [file ADVS-7-2001358-s001.pdf]

Copyright WILEY-VCH Verlag GmbH & Co. KGaA, 69469 Weinheim, Germany, 2020.

Supporting Information

Porous SnO_2/C nanofiber anodes and $LiFePO_4$/C nanofiber cathodes with a wrinkle structure for stretchable lithium polymer batteries with high electrochemical performance

O Hyeon Kwon, Jang Hyeok Oh, Bobae Gu, Min Su Jo, Se Hwan Oh, Yun Chan Kang, Jae-Kwang Kim, Sang Mun Jeong*, Jung Sang Cho**

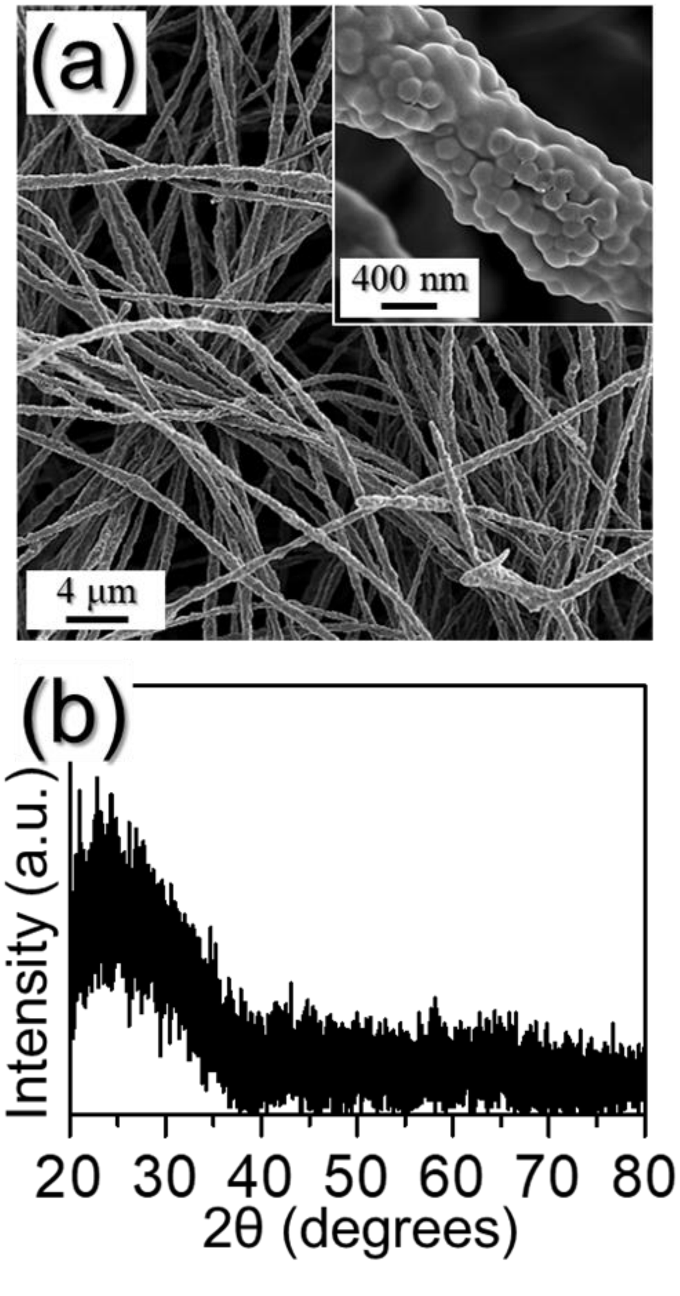

Figure S1. (a) FE-SEM image and (b) XRD pattern of the as-spun nanofibers for anodes composed with $SnCl_4 \cdot 5H_2O$, PVA, and PS nanobeads.

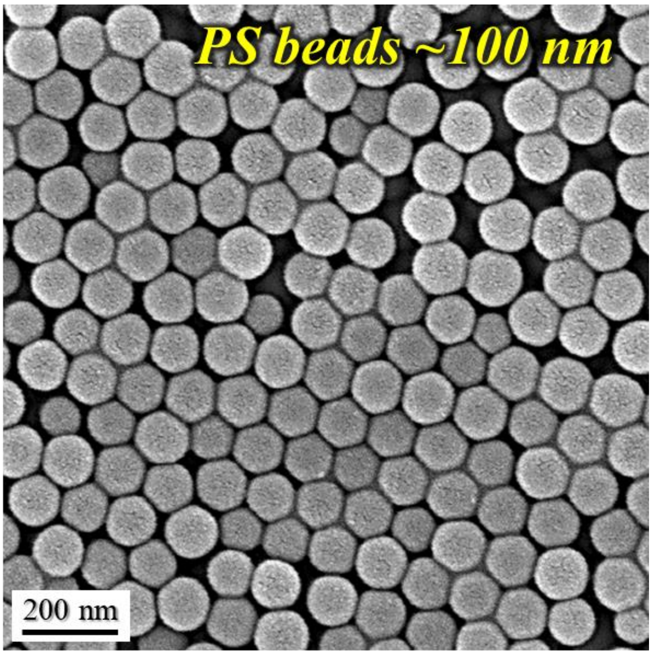

Figure S2. FE-SEM of the PS nanobeads with a diameter of 100 nm prepared by emulsion polymerization method.

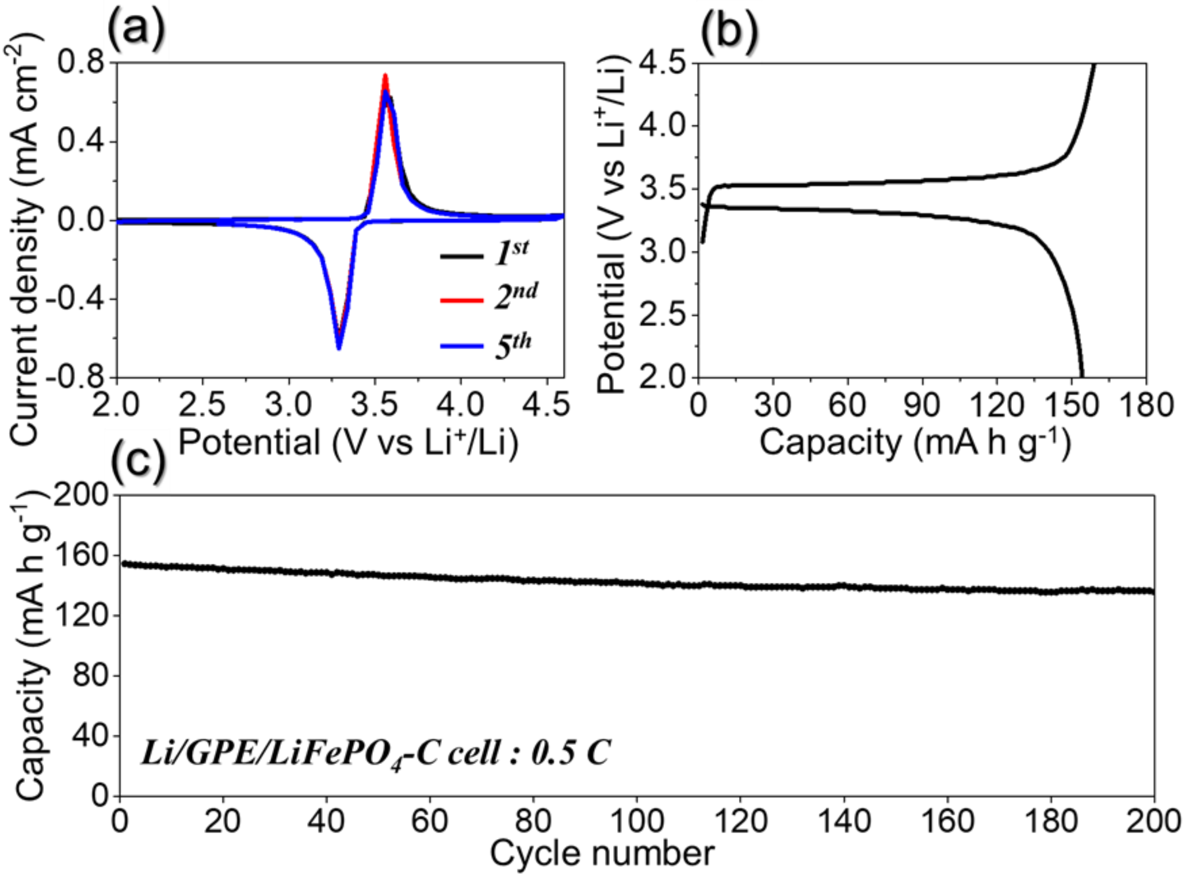

Figure S3. Electrochemical properties of the LFP/C NF as cathodes: (a) CV curves, (b) initial charge-discharge profile, and (c) cycling performance at a current density of 0.5 C

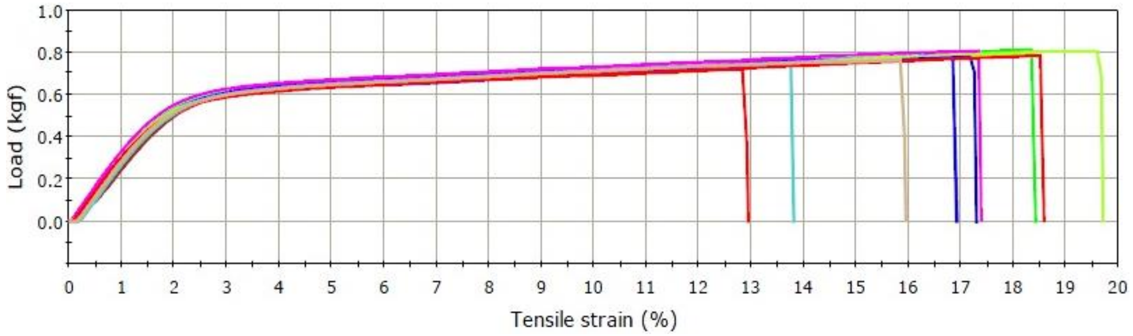

Figure S4. Tensile strength of the PVdF-HFP-based gel polymer electrolyte.

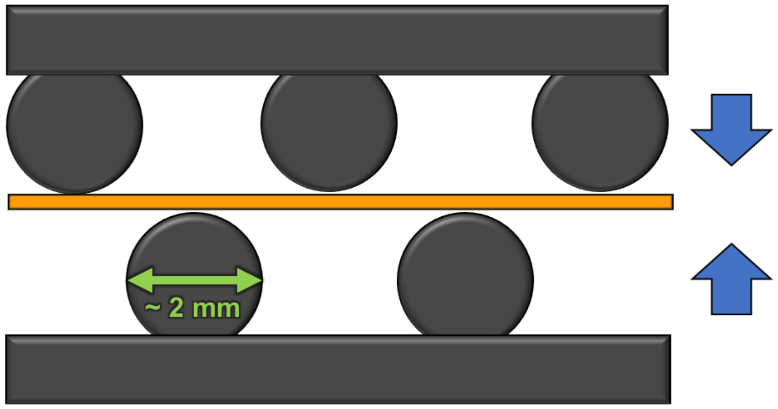

Figure S5. Schematic illustration of molding for the fabrication of wrinkled-type electrodes.

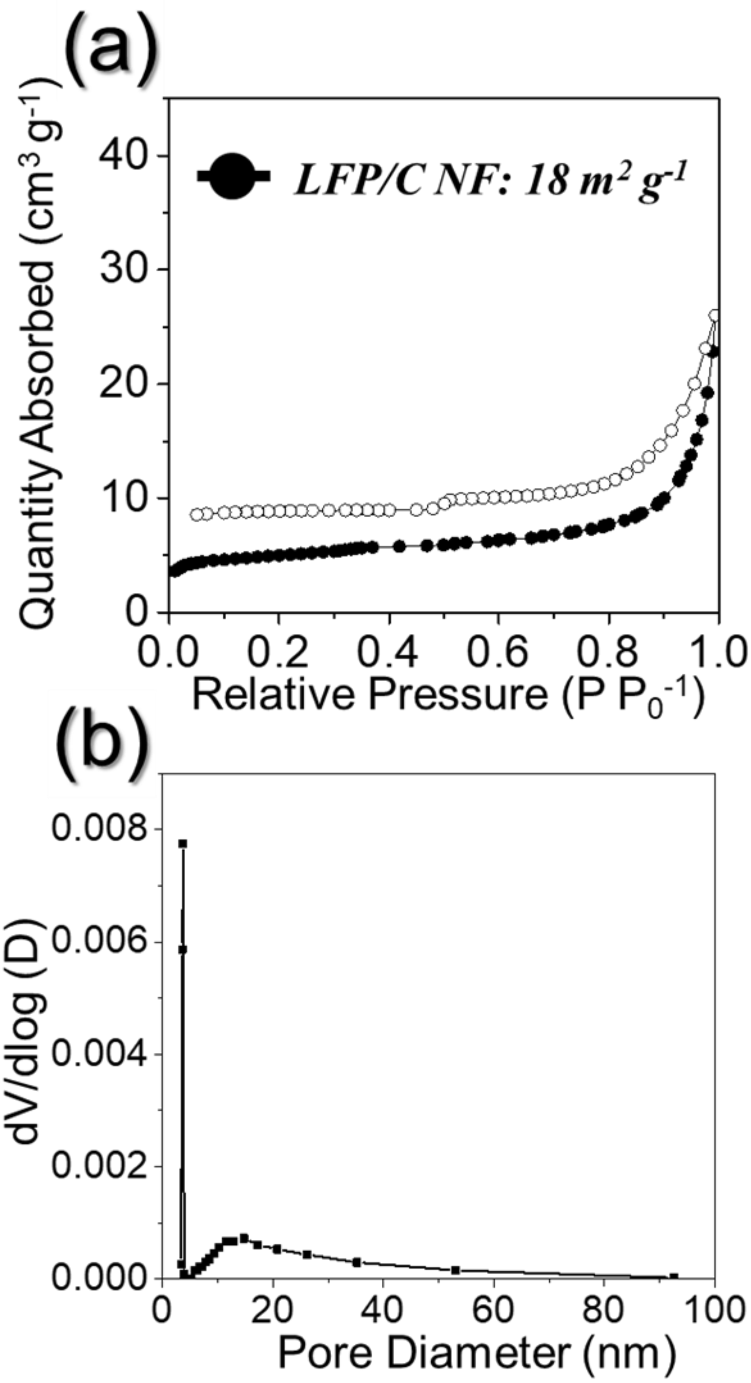

Figure S6. (a) N_2 adsorption and desorption isotherms and (b) pore size distribution of LFP/C NF.

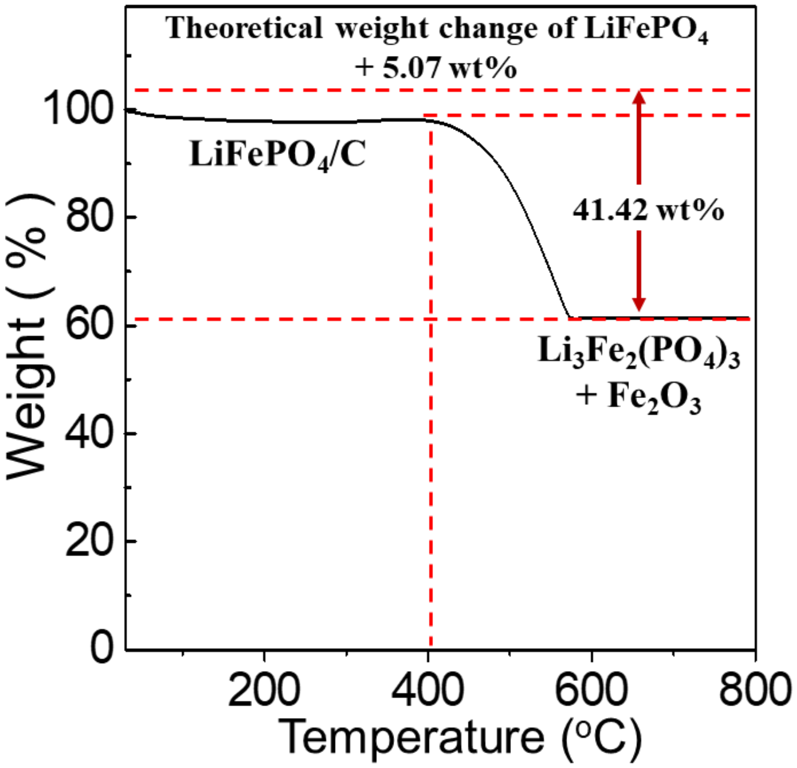

Figure S7. TGA curve of LFP/C NF.

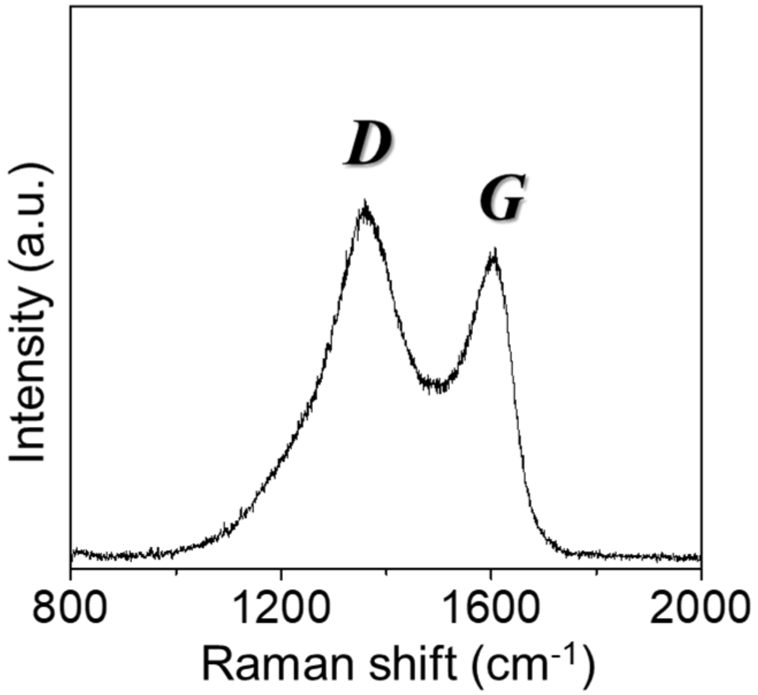

Figure S8. Raman spectroscopy of LFP/C NF.

Table S1. Comparison of the performances of stretchable batteries

Materials	Stretchable Strategies	Electrochemical properties under tensile strain	Energy density	Ref.
LFP/C@SnO$_2$/C	***Wrinkle structure***	***92% capacity retention***	***(unstretched) 458.8, (stretched) 423.4 Wh kg^{-1}***	***In this work***
LCO/graphite	Wavy structure	85% capacity retention	(unstretched) 110 Wh L^{-1}	[4]
LCO/graphite	Accordion-like design	96% capacity retention	(stretched) 233 Wh L^{-1}	[13]
LTO	3D porous sponge-like PDMS scaffolds	94% capacity retention	(unstretched) ≈180 Wh kg^{-1}	[15]
LCO/graphite	Kirigami structure	85% capacity retention	(unstretched) 160 Wh kg^{-1}	[16]
LMO/CNT@PI/AC	Jabuticaba-inspired hybrid carbon filler/polymer	80% capacity retention	(unstretched) ≈100 Wh kg^{-1}	[24]
LMO/CNT@PI/CNT	GAP multilayer conductor	72% capacity retention	(unstretched) ≈120 Wh kg^{-1}	[59]
LMO/CNT@MnO$_x$/CNT	Wrinkle structure	88% capacity retention	(unstretched) ≈291 Wh kg^{-1}	[S1]
CNT/LMO@CNT/LTO	CNT fiber spring	85% capacity retention	-	[S2]
ε-Li$_x$V$_2$O$_5$/LiMn$_2$O$_4$	SEBS/CNT/CB/Ag membrane	65% capacity retention	(unstretched) 35, (stretched) 17 Wh kg^{-1}	[S3]
Graphene-CNT/LFP@ Graphene-CNT/LTO	Re-entrant micro-honeycomb electrodes	94% capacity retention	(unstretched) 102.4 Wh kg^{-1}	[S4]

References

[S1] T. Gu, Z. Cao, B. Wei, *Adv. Energy Mater.* **2017**, *7*, 1700369.

[S2] K.-W. Kim, H. W. Kim, Y. Kim, J.-K. Kim, *Electrochim. Acta* **2017**, *236*, 394.

[S3] X. Chen, H. Huang, L. Pan, T. Liu, M. Niederberger, *Adv. Mater.* **2019**, *31*, e1904648.

[S4] S. Kang, S. Y. Hong, N. Kim, J. Oh, M. Park, K. Y. Chung, S. S. Lee, J. Lee, J. G. Son, *ACS Nano* **2020**, *14*, 3660.
